# Femtosecond laser‑induced herringbone patterns

**DOI:** 10.1007/s00339-018-1822-z

**Published:** 2018-05-02

**Authors:** Erik M. Garcell, Billy Lam, Chunlei Guo

**Affiliations:** 1 The Institute of Optics, University of Rochester, Rochester, NY 14627, USA; 2 Changchun Institute of Optics, Fine Mechanics, and Physics, Changchun 130033, China

## Abstract

Femtosecond laser-induced herringbone patterns are formed on copper (Cu). These novel periodic structures are created following s-polarized, large incident angle, femtosecond laser pulses. Forming as slanted and axially symmetric laser-induced periodic surface structures along the side walls of ablated channels, the result is a series of v-shaped structures that resemble a herringbone pattern. Fluence mapping, incident angle studies, as well as polarization studies have been conducted and provide a clear understanding of this new structure.

## 1 Introduction

Herringbone patterns are arrangements of nested v-shaped structures, so called for their resemblance to the skeleton of a herring fish. In addition, as seen in nature in the branching veins of some leaves and the flight feathers of birds, herringbone structures are excellent biomimetic surfaces researched for their ability to increase structural stability of materials [1], reduce drag on surfaces [2], and induce transverse flow in microchannels [3, 4]. In this work, using pulsed femtosecond laser irradiation, we have developed a rapid, single step, technique for the production of herringbone structured surfaces. These structures form as slanted periodic grooves on the sides of a central channel or backbone. These grooves show similarities to laser-induced periodic surface structures (LIPSSs). LIPSS, otherwise known as surface ripples or nanogratings, are parallel periodic grooves produced on the surface of materials by laser irradiation, having a period proportional to the laser’s wavelength [5, 6]. First observed by Birnbaum in 1965 on Ge [7], LIPSS have since been extensively studied using both continuous and relatively long-pulsed lasers [8–12]. More recently, LIPSS have been investigated using femtosecond lasers on a variety of materials, including semiconductors and dielectrics by others [13–17], and on metals by us [18–22]. Several theories exist, and the generally accepted means by which these structures are formed is by the interference of incident laser light with surface scattered electromagnetic waves, causing periodic modulation of the laser’s deposited energy, resulting in inhomogeneous ablation of material [23–25].

In this paper, we report the observation and study of a new type of LIPSS, named here as herringbone LIPSS, as these structures closely resemble a herringbone pattern. Instead of having a uniform symmetry, the herringbone LIPSSs we produce have axial symmetry along the channel they form along and are orientated at an acute angle to the polarization, as opposed to standard LIPSSs, which tend to form either perpendicular or parallel to the laser’s polarization [26]. Spatially modulated LIPSSs have been observed by others before, by producing periodic structures with differing orientation in different spatial locations [21, 27, 28]. However, unlike other spatially modulating LIPSS structures, herringbone LIPSSs are produced using only a single beam and form with axially symmetry. To characterize the structures we produced, we conducted a series of experiments that examined influences of angle of incidence, laser fluence, and polarization direction to fully diagnose the mechanisms responsible for the evolution and origin of this novel structure.

## 2 Methods

**Fig. 1:**
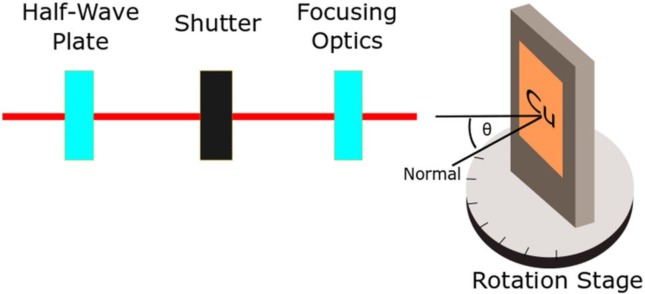
Experimental setup used for herringbone pattern studies

Experiments were carried out using a pulsed Ti–sapphire femtosecond laser system that generates 50 fs linearly polarized pulses operating at a repetition rate of 1 kHz at a central wavelength of 800 nm. The laser beam polarization was varied using a half-wave plate. The sample material used in these experiments was polished, oxygen-free, high thermal conductivity Cu. The number of pulses was selected using an electromechanical shutter. Samples were mounted vertically on a rotation stage to alter the laser beam’s incidence angle on the sample (Fig. 1). The laser beam was focused upon the Cu surfaces using a 200 mm focal distance, plano-convex lens. The surface structures of the irradiated samples were studied using a scanning electron microscope (SEM) and a UV laser-scanning confocal microscope (UV-LSCM). All images shown were taken at normal incidence to the Cu surface, and oriented with the laser beam traveling towards the right-hand side of the image. All data generated or analysed during this study are included in this published article.

Absorption varies with polarization and incident angle according to the Fresnel equations [29]. To maintain constant fluence across irradiation parameters within an experimental set, we varied the laser beam’s power to both compensate for the change in Fresnel absorption and crosssectional area whenever the polarization or incident angle was altered. For Cu, a refractive index of 6.12 × 10^−2^ and an extinction coefficient of 5.13 were used to calculate the Fresnel absorption [30].

## 3 Results

**Fig. 2:**
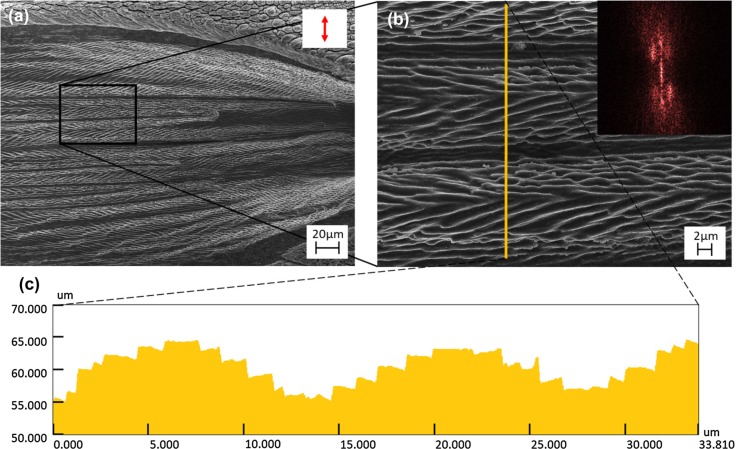
**a** Image of herringbone LIPSS formed on Cu at a 50◦ angle of incident, following 20 K pulses at a single pulse fluence of 0.25 J/cm^2^, taken with a UV laser-scanning confocal microscope. The arrow shows the orientation of the incident polarization. **b** Magnified view with a corresponding 2D-FFT of the image shown in the top right. **c** Surface profile for the line shown in image **b**

Figure 1 shows typical herringbone LIPSSs. These structures are created at large incident angles and with s-polarized light. Femtosecond laser-induced herringbone structures form as periodic LIPSSs along the walls of ablated channels, dug into the sample’s surface (Fig. 2a, b). Channels, in this case, are referring to long and continuous ablated regions having a pair of sidewalls resembling a trough-like structure (Fig. 2c). The LIPSSs forming on the channel sidewalls are angled outward with respect to the laser’s propagating direction and have axial symmetry along the channel. The resulting pattern is a series of v-shaped structures that resemble a herringbone pattern.

### 3.1 Fluence mapping

**Fig. 3:**
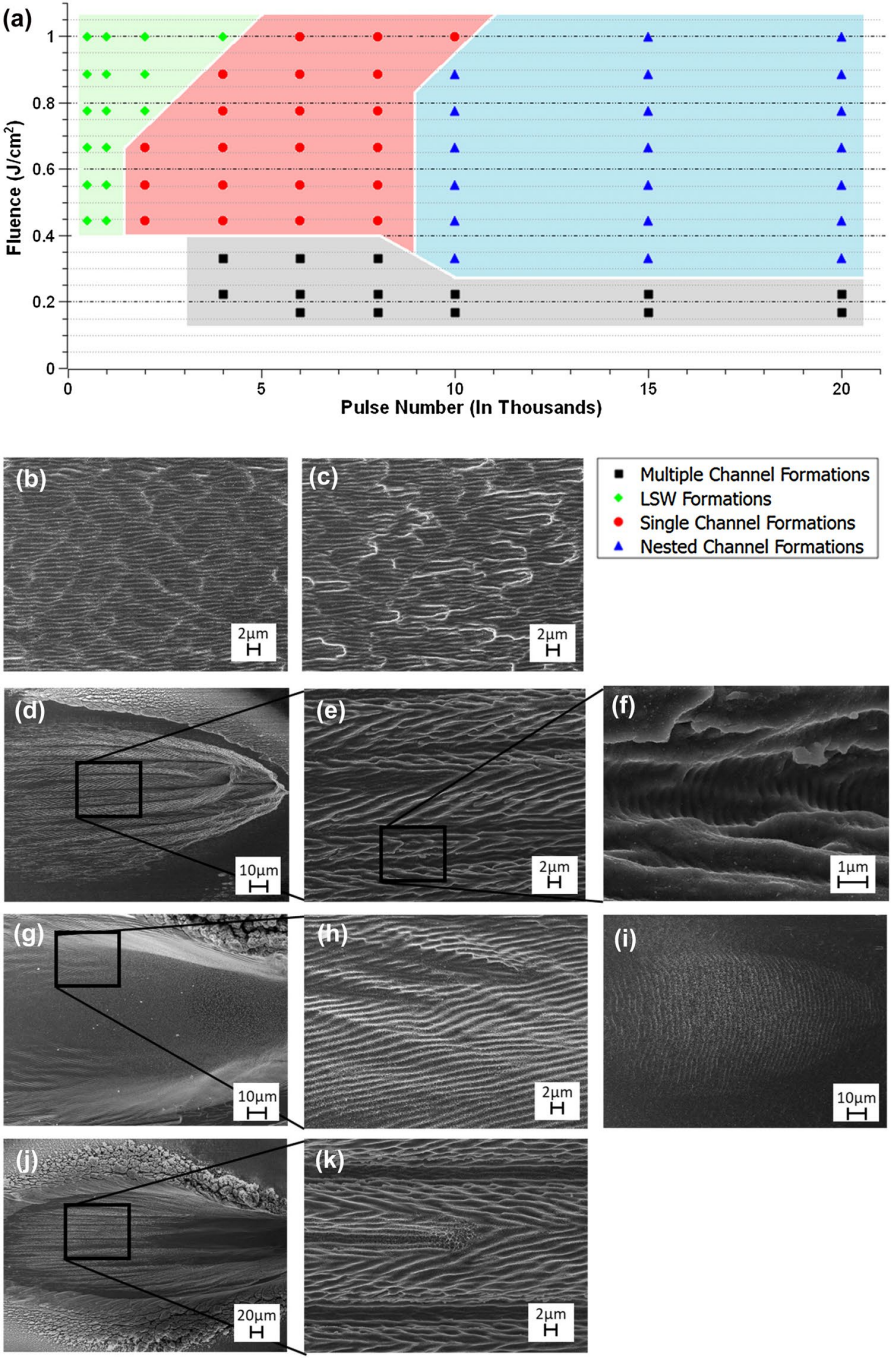
Fluence map and SEM images of Cu irradiated by a pulsed femtosecond laser at a 50◦ incident angle. **a** Graph of the fluence zones of various structures related to herring-bone LIPSSs. **b** Standard LIPSS formations following 500 pulses at a fluence of 0.22 J/cm^2^. **c** Initial channel formation following 1 K pulses at a fluence of 0.17 J/cm^2^. **d** Representative example of the multiple channel formations fluence band taken at a fluence of 0.17 J/cm^2^ following 20 K pulses with **e** being herringbone structures and **f** HSFL within this fluence band. **g** Single channel formations fluence band taken at a fluence of 0.66 J/cm^2^, after 8 K pulses. **h** Undulation of LIPSS structures on the sidewalls of the singular channel formations. **i** LSW formations occurring at 0.53 J/cm^2^ following 500 pulses. **j** Image of nested channel formations fluence band taken at a fluence of 0.45 J/cm^2^ with 20 K pulses. **k** Bifurcation occurring within herringbone LIPSS formations

To study this unique laser-induced formation, we have mapped out in detail the fluence regimes for which we have produced herringbone LIPSS structures (Fig. 3a). For this study, all shots were taken at a 50◦ incident angle, using s-polarized light. Single pulse fluence was varied from 0.1 to 1.0 J/cm^2^ and pulse train number was varied from 500 to 20 K. Careful investigation of surface structures produced, following variations in laser fluence and pulse number, demonstrates the presence of three distinct fluence zones, where herringbone LIPSSs can be formed. These fluence regions are categorized on the basis of geometry of channel structures produced and are named multiple channel formation, single channel formation, and nested channel formation fluence zones.

For relatively low fluences, initial structures observed are standard LIPSS, otherwise known as low spacial frequency LIPSSs (LSFL), oriented perpendicular to the laser’s polarization with an average periodicity of 800 nm, predominantly forming at a single pulse fluence value of 0.1 J/cm^2^ (Fig. 3b). LSFL are characterized as having a periodicity roughly equal to the inciting laser’s wavelength [6]. As fluence is increased to between 0.1 and 0.15 J/cm^2^, short and shallow channels form amongst the LSFL (Fig. 3c). With increasing fluence, these short channels grow in length and depth, transitioning into the multiple channel formation fluence zone. Multiple channel formations are formed for fluence values in the band of 0.15–0.3 J/cm^2^. This fluence band is characterized by having a multitude of densely packed, long and shallow channels covered in periodic herringbone LIPSSs (Fig. 3d, e). On and between the herringbone LIPSS formations formed in this fluence band, high spatial frequency LIPSS (HSFL) can be seen forming with a periodicity of 360 nm (Fig. 3f). HSFL are LIPSS structures having a period roughly half that of the inciting laser’s wavelength and can be found forming either parallel or perpendicular to the laser’s polarization [31, 32].

Single channel formations, the second fluence region where femtosecond laser-induced herringbone structures are formed, are located in a fluence band above 0.4 J/cm^2^, and for pulse numbers between 1 and 10 K. This fluence band is characterized by a single angled hole drilled into the material’s surface. Beginning at 1 K pulses, the laser-drilled pit is shallow and has only slightly raised sidewalls. As pulse number increases, continued ablation deepens the pit into a hole or channel and elongates the channel into the material’s surface, forming large curved sidewalls where angled LIPSSs form (Fig. 3g). This singular channel with angled and axially symmetric LIPSSs constitutes a herringbone structure. The LIPSSs forming along the curved sidewalls of the single ablated channel, can be observed undulating with the changing sidewall angle (Fig. 3h).

Also forming for fluences in excess of 0.4 J/cm^2^, but for pulse numbers below 4 K, large scale waves (LSWs) can be observed (Fig. 3i), and have been observed by us previously [33, 34]. LSWs are periodic surface structures, resembling sand dunes in the desert. These structures have periods tens of times greater than the incident laser wavelength, and are formed from periodically distributed nonuniform heating following pulsed irradiation.

Nested channel formations, the third and final fluence region, where we have created herringbone LIPSSs, are formed at fluences above 0.3 J/cm^2^, and for pulse numbers in excess of 8 K. Instead of forming only a single laser-drilled hole as would form for lower pulse numbers at these fluences, separate inner channels grow within the larger ablated pit. Inner channels can span the entire length of the ablated pit and are typically covered with herringbone LIPSSs (Fig. 3j). Bifurcation, a common phenomenon in LIPSS formations where a singular regularly-oriented ripple formation can be seen bisecting [35], has been observed occurring along herringbone LIPSS formations within this fluence zone (Fig. 3k).

Across the three fluence zones, the periodic grooves that form herringbone LIPSSs are observed forming at angles between 5 ◦ and 30◦ , relative to the axis of the channel they form along. Measuring parallel to the channel axis, these same herringbone structures are observed forming with periodicities between 4 and 8 μ m. These measurements were taken with with respect to the plane of the sample.

### 3.2 Polarization dependence

When producing herringbone LIPSSs, it is observed that they only form for s-polarized laser irradiation. To understand this structure’s dependence on s-polarization, a study was performed at an incident angle of 50◦ , traversing polarization from 0 ◦ (p-polarized) to 90◦ (s-polarized), in 10◦ increments. For each polarization, data were taken at a pulse train value of 15 K, using an absorbed single pulse fluence of 0.25 J/cm^2^. Careful attention was paid to maintain a consistent fluence value as absorptance changed with polarization. The selected fluence value corresponds to the multiple channel formations fluence band previously observed for s-polarized irradiation in the fluence mapping study. This fluence was selected for the clear and uniform herringbone structures capable of being produced.

**Fig. 4:**
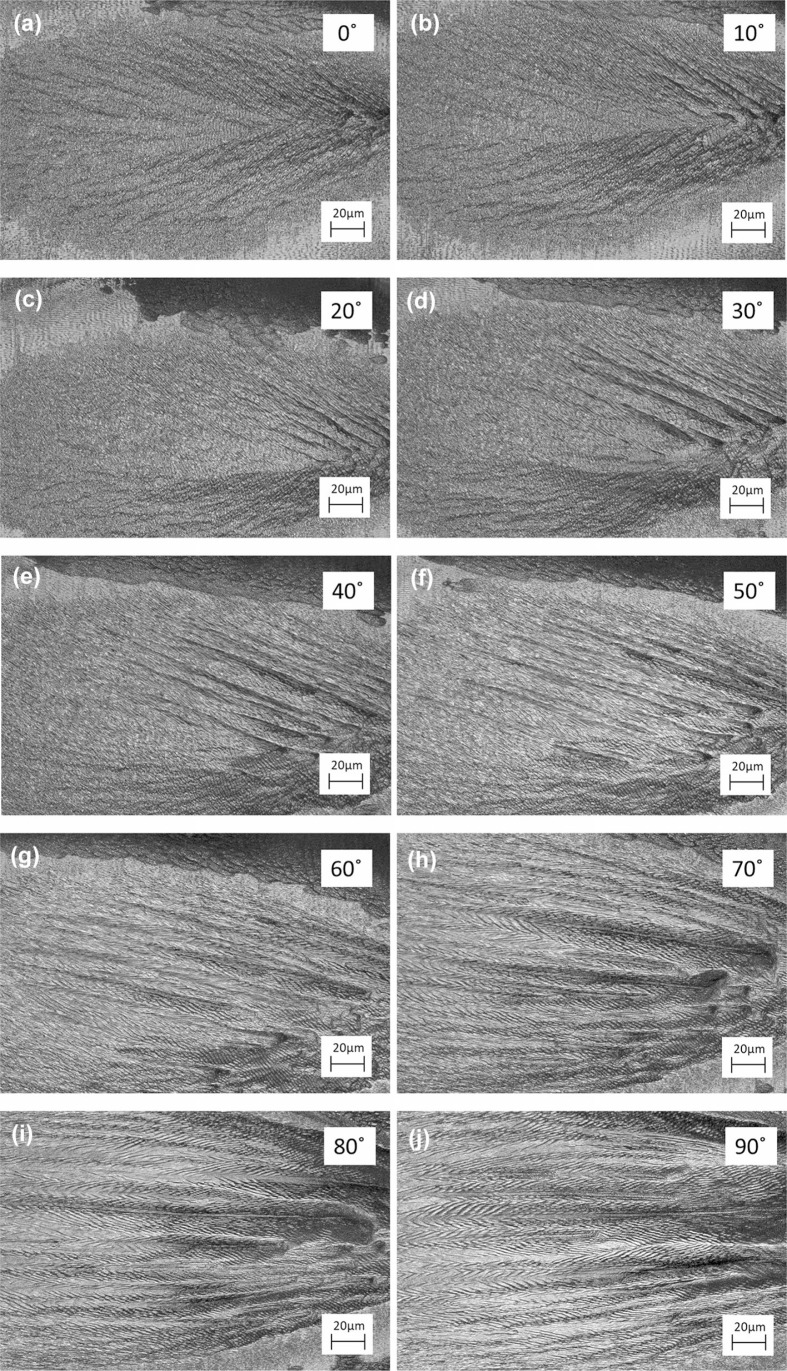
Images of Cu irradiated at varying polarizations (indicated in the top right), at a 50◦ angle of incidence, with a 15 K pulse train at a single pulse fluence value of 0.25 J/cm^2^

The resulting images contain two structures: LIPSS and large wall structures which increase in size and number with increasing laser pulse number (Fig. 4). The wall structures are oriented differently than the underlying LIPSS structures but trend in the same manner, with increasing polarization causing increasing like-rotation of the walls. At 0 ◦ polarization, the walls formed are roughly oriented at an angle 45◦ to the underlying LIPSS (Fig. 4a), but as polarization increases this angle approaches parallel. At approximately 30◦ polarization, the walls pack themselves more closely, forming channels between them (Fig. 4d). As polarization continues to become more heavily s-polarized, these channel walls grow in length. Only when polarization approaches 80◦ polarization does one see the creation of symmetric or near-symmetric LIPSS structures occurring on the channel walls, forming herringbone LIPSSs (Fig. 4ij).

### 3.3 Incident angle dependence

In addition to being polarization restricted, these structures are only formed at large incident angles. An additional study was conducted to determine the effects of the laser’s incident angle on the formation of herringbone LIPSSs. For this study, the incident angle of the laser with respect to the Cu sample was varied in 5 ◦ steps from 0 ◦ to 55◦. The study was performed using s-polarization and an absorbed single pulse fluence of 0.20 J/cm^2^. Careful attention was paid to maintain this fluence consistently across all incident angles, as material absorptance and cross-sectional area changes with incident angle. As was the case for the polarization dependence study, the selected fluence value for this study corresponds to the multiple channel formations fluence band observed for 50◦, s-polarized, irradiation. This fluence was chosen for the multiple clear herringbone patterned channel formations capable of being produced.

**Fig. 5:**
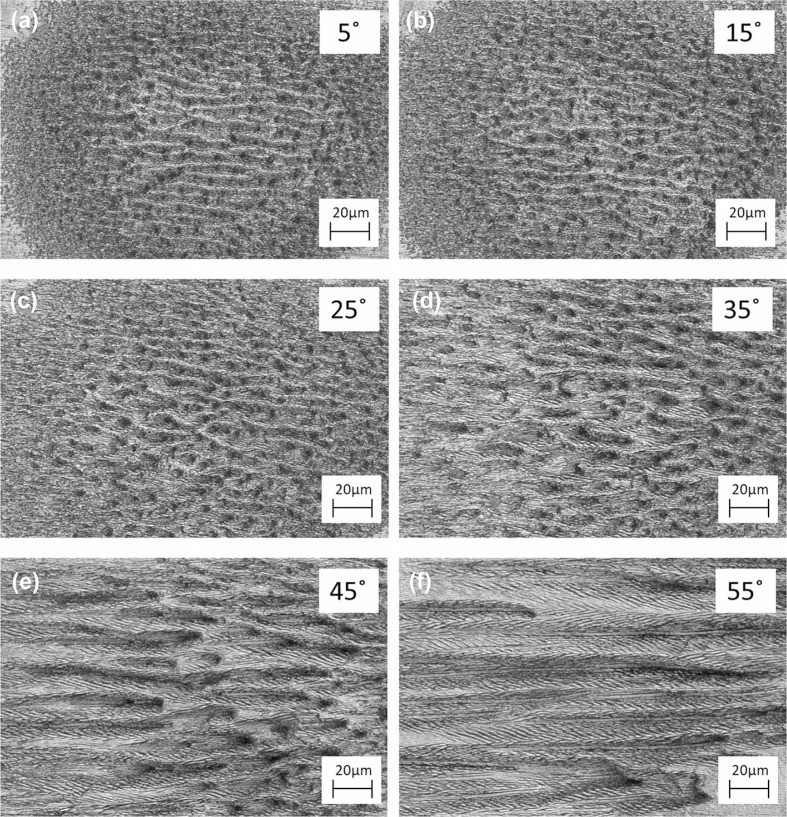
Images of Cu irradiated at varying incident angles, at s-polarization, with 10 K pulses at a single pulse fluence value of 0.20 J/cm^2^

At normal incident, the entirety of the ablated area is covered in LSFL structures with an average period of 800 nm, and pits formed by defect-focused ablation (Fig. 5a). At incident angles between 0 ◦ and 45◦ , these pits grow out and form shallow, closely packed channels; LIPSSs formed in these channels are irregular (Fig. 5b). Between 45◦ and 55◦ , channels grow further in length and, for these incident angles, LIPSSs formed along the channel walls now form into orderly, symmetric, herringbone LIPSSs (Fig. 5e, f).

## 4 Discussion

The herringbone LIPSSs here formed are bounded by a number of variables including pulse train amount, single pulse fluence value, angle of incident, and polarization. Interestingly, it is observed that good quality herringbone patterned LIPSSs can only be fabricated using s-polarized light (± 10◦). Deviation from this polarization will cause LIPSSs forming on channel walls to be asymmetric. In addition to polarization considerations, channels of significant depth and length need to be formed to form herringbone structures along the channel walls. This requires a minimum 35◦ incident angle from normal and a threshold fluence and pulse train amount, for 50◦ incident angle ablation, that translates to between 0.15 and 0.17 J/cm^2^ and 3–4 K pulses.

Herringbone LIPSSs share many characteristics with standard LIPSSs; beyond visual similarities, both being periodic surface structures, herringbone LIPSSs also exhibit bifurcation of structures similar to what has been observed for other LIPSS structures [35]. Herringbone structures also form alongside both low and high spatial frequency LIPSSs, illustrating that the fluences needed to form herringbone LIPSS overlap with those of standard and frequency doubled LIPSSs. However, herringbone LIPSSs differ from other LIPSS structures in that they form with axial symmetry, exhibiting a sharp discontinuity in their structure, and form with periodicities many times greater than the wavelength of the inciting laser. LIPSS typically form uniformly across the surface of a material and have a periodicity roughly equal to the laser’s wavelength [6]. Though several theories exist as to the formation of LIPSSs, it has been shown that LIPSS formations are highly dependent on polarization direction, forming predominantly perpendicular to the surface projection of the laser’s polarization [26, 36]. Assuming herringbone LIPSS form similarly to standard LIPSSs, the slope of the ablated sidewalls they form along would cause the projection of the laser beam’s polarization onto this new incident plane to be different from the projected orientation on the flat surface. These angled channel sidewalls are clearly, as shown in Fig. 1c. With the surface component of the polarization altered on channel sidewalls, LIPSS forming on channel sidewalls will likewise be altered.

**Fig. 6:**
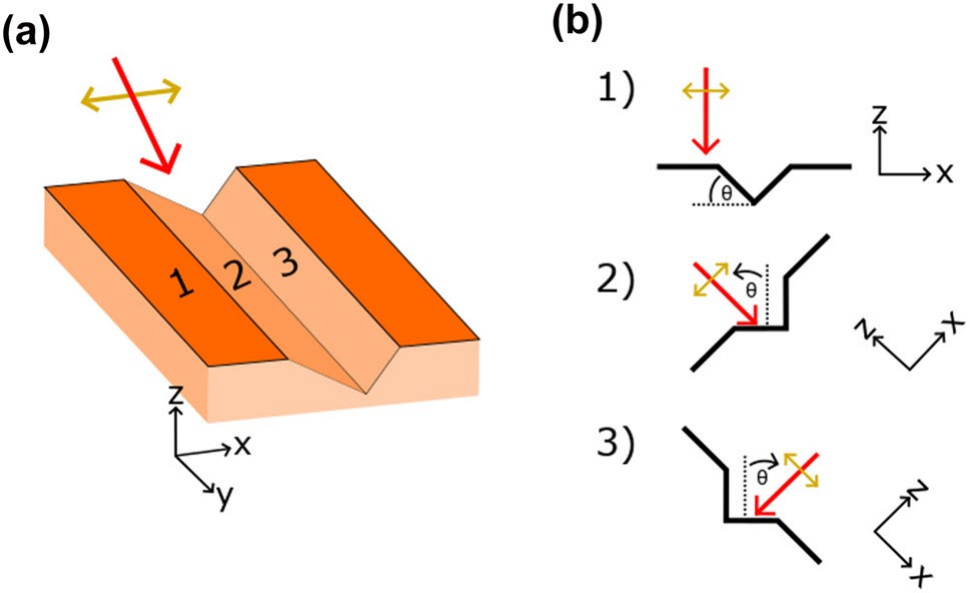
Graphic illustrating the change in polarization on the sidewalls of ablated channels irradiated by an s-polarized laser beam. The single and double headed arrows represent the incident laser beam and its polarization, respectively. **a** Material with a groove cut into its surface. Surface 1 is flat, while surfaces 2 and 3 are sloped and oppositely oriented. **b** Slices of image a, showing the change in polarization in the reference of sloped surfaces 2 and 3

As our studies have demonstrated, herringbone LIPSSs are formed by irradiation with s-polarized light at large incidence angles (≥ 35◦). After initial irradiation with these conditions, and at sufficient fluences, defect-focused ablation is observed forming small pits on the material’s surface that then grow out and form dug-out channels upon further irradiation. The s-polarized non-normal incident laser light contacting the angled channel walls has an altered plane of incidence and consequently an altered projected polarization, with respect to the plane of the sample (Fig. 6). Since LIPSSs orientation changes proportionally with a change in polarization of the incident light, LIPSSs formed on the channel walls will have an altered orientation compared to LIPSSs formed on the plane of the sample. Assuming the walls of the channel form with axial symmetry, the LIPSSs formed on the channels will also form symmetrically, thus creating herringbone patterned LIPSSs.

## 5 Conclusion

In conclusion, we have formed and studied femtosecond laserinduced herringbone LIPSSs on Cu. Several experiments were conducted to characterize and better understand the formation mechanisms of this new feature. A mapping of fluence space illustrated the various regimes in which herringbone LIPSS structures have been formed, while polarization and incidence angle studies placed bounds on the parameters necessary for their formation. Studies find that these v-shaped periodic structures require fluence and pulse numbers high enough to ablate long, dug-out channels along the material’s surface. Once channels are present, the altered projection of the laser beam’s polarization on the sidewalls of laser ablated channels causes local modulation in the orientation of LIPSSs formed on said sidewalls. For symmetrically formed channels, the modulation in the laser beam’s polarization, and consequently the orientation of LIPSSs, will have reflected symmetry across the two walls of the channel, creating the appearance of v-shaped structures. The herringbone LIPSSs, here formed and characterized, can see application in a great number of fields, such as bio-analytics, synthetic chemistry, aviation technologies and many others, as herringbone structures are important and extensively studied structures in both microfluidics mixing [37, 38] and biomimetic surfaces [2, 39].
